# Aphid Species in Citrus Orchards in Crete: Key Vectors of Citrus Tristeza Virus and Automated Monitoring Innovations for Alate Aphids

**DOI:** 10.3390/v17030395

**Published:** 2025-03-11

**Authors:** Matthaios M. Mathioudakis, Kyriaki Varikou, Antonia Karagianni, Panagiota Psirofonia, Nikolaos Tektonidis, Despoina Kapantaidaki, Vasiliki Evangelou, Leonidas Economou, Beata Hasiów-Jaroszewska, Ilyas Potamitis

**Affiliations:** 1 Plant Pathology Laboratory, Institute of Olive Tree, Subtropical Crops and Viticulture, ELGO-DIMITRA, Karamanlis Ave. 167, 73134 Chania, Crete Island, Greece; tektonidis@elgo.gr; 2 Department of Entomology, Institute of Olive Tree, Subtropical Crops and Viticulture, ELGO-DIMITRA, Karamanlis Ave. 167, 73134 Chania, Crete Island, Greece; varikou@elgo.gr (K.V.); karagianni@elgo.iosv.gr (A.K.); 3 Department of Music Technology & Acoustics, Hellenic Mediterranean University, 74100 Rethymno, Crete Island, Greece; ppsirof@hmu.gr; 4 Laboratory of Agricultural Entomology Scientific Directorate of Entomology and Agricultural Zoology, Benaki Phytopathological Institute, St. Delta 8, 14561 Kifissia, Attica, Greece; debora-kap@hotmail.com (D.K.); v.evangelou@bpi.gr (V.E.); 5 Scientific Directorate of Pesticides Control and Phytopharmacy, Benaki Phytopathological Institute, St. Delta 8, 14561 Kifissia, Attica, Greece; l.economou@bpi.gr; 6 Institute of Plant Protection-National Research Institute, Department of Virology and Bacteriology, ul. Wł. Węgorka 20, 60-318 Poznań, Poland; b.hasiow@iorpib.poznan.pl

**Keywords:** aphid, transmission, CTV, citrus, mtCOI, RT-PCR, optoelectronic sensors, artificial intelligence, viruliferous aphid species

## Abstract

*Citrus tristeza virus* (CTV) is a vector-borne virus that poses a significant threat to citrus production worldwide, inducing a variety of symptoms. Therefore, a detailed knowledge of local aphids, identification of viruliferous species, and the development of new monitoring tools are necessary to improve CTV control strategies. Herein, a 2-year survey was conducted to assess the frequency of aphid species infesting several citrus pilot orchards. Plot findings based on morphological and molecular identification revealed *Aphis spiraecola* (ranged from 44–100%) as the most abundant aphid species, followed by *A. gossypii* (<50%). *Toxoptera aurantii, Myzus persicae*, and *A. craccivora* were present in low numbers, and *A. citricidus* was not detected. Due to the absence of CTV detection in aphids and citrus trees from the pilot orchards, a complementary survey was conducted in CTV-infected fields. Three aphid species were identified as CTV-positive by RT-PCR, suggesting that they may be viruliferous, with *A. spiraecola* as predominant, followed by *A. gossypii* and *T. aurantii*. Additionally, we developed a non-invasive procedure for identifying aphid species using wingbeat analysis. This method provides a faster alternative to traditional identification techniques by taxonomic keys based on morphological features or PCR, although its accuracy is lower (approximately 95% for the two species tested). Overall, this work provides a detailed study of aphid species composition in citrus orchards, identifies the predominant local putative CTV vector, and introduces a novel sensor for aphid monitoring, contributing to improved epidemic forecasting and sustainable disease management strategies.

## 1. Introduction

Citrus species represent a globally significant agricultural commodity, with annual production > 140 million tons [1], thereby establishing a substantial source of income. In Greece, citriculture, with more than 19 million trees predominantly located in the southern and northwestern parts of the country (mainly in Crete and Peloponnese), is one of the three most important fruit crops, highlighting its immense social-economic importance [2]. However, citriculture is threatened by the occurrence of pests and vector-borne viral diseases, which can compromise the final product quality and increase input requirements [3]. Therefore, accurate identification of pests and their dynamics, as well as their potential to serve as vectors for diseases, are necessary to sustain healthy crops and to develop control strategies.

Aphids typically appear during the citrus growing season from late spring to early summer and late summer to early autumn, coinciding with periods of tender flush growth. Aphids can cause both direct damage, such as leaf deformation, and indirect damage, like the development of sooty molds in honeydew secretions, and predominantly transmit viral pathogens on citrus trees, like citrus tristeza virus (CTV) [4]. While all aphid species can acquire and transmit the CTV in a semi-persistent manner, their efficacy varies depending on the aphid species and the virus strains [5].

*Aphis citricidus* (Kirkaldy), or brown citrus aphid, is considered one of the most damaging aphid species affecting citrus plants worldwide and the most important vector of CTV due to its high transmission efficiency [6,7,8]. *A. gossypii* (Glover), the cotton aphid, is also an efficient CTV vector in California, Israel, and Spain [9,10,11], while *Toxoptera aurantii* (Boyer de Fonscolombe) and *A. spiraecola* Patch are considered less efficient vectors [12]. Besides flush cycles affecting CTV transmissibility by the viruliferous aphids [5,13,14], the aphid population dynamics play a pivotal role in CTV transmissibility, which can vary even among similar orchards in a specific area. The factors that primarily determine the population dynamics of aphids are related to environmental parameters such as abiotic factors, host plant vigorousness, and aphid migration limitations. To date, previous studies in Greece have identified nine species of aphids [15,16,17] without any record of *A. citricidus*.

CTV is a typical member of the genus *Closterovirus* (family *Closteroviridae*) with a single-stranded RNA genome of approximately 19.3 kb consisting of 12 open reading frames [18]. CTV naturally infects citrus and other related genera, causing one of the most destructive citrus diseases worldwide [14,19]. Depending on the virus genotype and the scion–rootstock combination, CTV appears in three syndromes with variable severity and symptomatology: quick decline, stem pitting, and seedling yellows [14,20,21]. Currently, 11 distinct CTV genotypes have been identified based on whole genome sequences, which can exist individually or in combination as a complex within a citrus plant [22]. The introduction of CTV in new areas occurs through plant material exchange and aphid vectors, both of which have contributed to the vast expansion of CTV [14]. In Greece, various citrus viruses and viroids have been recently reported [23,24,25], and 20 years after the initial report of CTV in oranges in Crete and Peloponnese [26], it is still considered a quarantine pathogen that has gradually become endemic in various areas, along with the invasion of new hosts [27,28,29]. Three CTV genotypes have been detected so far in Greece: the severe VT genotype, restricted in northern areas, and the T30 (mild) and RB (severe) genotypes in Crete [27,28,29]. Notably, the identity of aphid species serving as local vectors for CTV transmission in Greece remains to be elucidated.

Identifying aphid species morphologically using a stereoscope has certain practical disadvantages. The process is time-consuming and requires specialized expertise, as distinguishing features require examination of high magnification of specimens of alate and apterous adult aphids, which sometimes can be damaged through sample preparation. Molecular identification requires a different type of expertise and equipment. The need for high-quality equipment and skilled personnel further limits accessibility, making the method less efficient for large-scale monitoring or rapid diagnostics. Because of these practical difficulties, new optical methods for monitoring insect species that utilize advanced light modulation and reflection techniques to capture unique wingbeat patterns [30,31,32] can be considered an optional method for aerial-borne insects like alate aphids. By analyzing these optical signatures, these systems offer non-invasive, real-time identification of insects in flight, aiding in environmental monitoring, pest control, and biodiversity research. Optical approaches, as applied to the open field or embedded in insect traps, enhance accuracy and automation in insect counting and identification. The captured optical signal stemming from a flying insect contains the wingbeat frequency and other characteristics, such as the amplitudes of the harmonics that can be shown to be characteristic of a specific species. The size and morphology of the insect, the way its flight-related muscles move the wings, and the frequency that beats its wing are a kind of biometric parameters. Despite the limitation of not being as accurate as morphological identification with keys or molecular identification, insect species recognition based on wingbeat patterns offers several advantages [30,33,34]. It can be non-invasive, allowing for the monitoring of insect populations without capturing or disturbing them. It does not require the termination of the insect specimen. It is relatively inexpensive and can be embeddable in insect traps so that insects are classified by artificial intelligence programs almost instantly [30]. It can also be deployed in remote or hard-to-reach locations to give proxies for the captured population. When optical means based on backscattered light from insects form arrays large enough to become stand-alone stations, they can give a rough distribution of flying insect fauna with sufficient classification accuracy [33].

Recent observations from multiannual monitoring of CTV in Crete at our laboratory suggest that a natural virus spread is occurring, necessitating detailed studies. The present work aims to determine the aphid population distribution in citrus orchards and species composition and identify prevalent aphid species as CTV vectors. This information is critical for developing optical devices capable of clearly detecting specific aphids in flight and registering their wingbeat for surveillance and monitoring, allowing automated quantification of the risk to a crop. These results enrich our knowledge of aphid dynamics and identification of putative viruliferous vectors, facilitating the development of sustainable approaches for the control of both aphids and CTV.

## 2. Materials and Methods

### 2.1. Aphid Samplings and Species Identification

**Figure 1:**
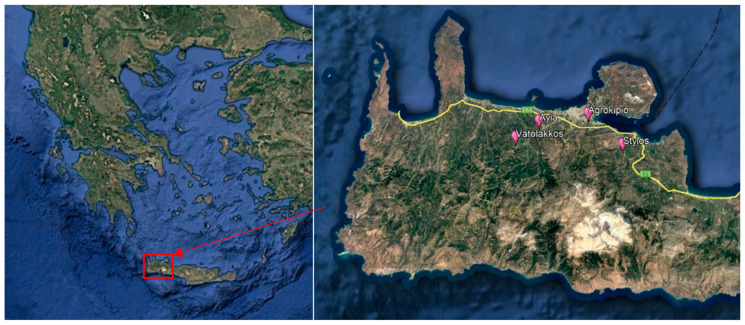
Experimental orchards are located in the western area of Crete Island, Chania prefecture, Greece (Source: Google Earth). The **left** panel shows the map of Greece, and in Crete, the red square is the area of Chania where the four pilot experimental orchards are located (**right** panel; in magnification).

Surveys were conducted in 10 pilot orchards, 15 to 20 years old, located in four citrus-producing areas of Chania prefecture (Vatolakkos, Ayia, Apokoronas (Stylos), Agrokipio), in western Crete, Greece (Figure 1, Table 1). During the sampling periods, insecticide applications were excluded; only standard agricultural practices such as pruning, irrigation, fertilization, and weed management were implemented. Samplings were performed from the autumn of 2020 (one sampling event) through the spring of 2022 (Table 1). Citrus stem samples were collected from the selected orchards at 10 to 15-day intervals, resulting in 14–19 sampling events per orchard (Table 1). This extensive sampling was undertaken to assess the presence of established aphid colonies on tender new vegetation. From each selected orchard, all aphid-infested tender shoots (1–4 stems/tree), each with a minimum of six leaves and a length of 10 cm, were collected from up to 20 trees. The collected shoots were then placed in sealed plastic bags with moistened paper towels to maintain humidity within a Styrofoam box. On each sampling date, individuals infesting each shoot were counted (nymphs, alate and apterous adults), and the adults were identified to species in laboratory conditions under a stereoscope (Carl Zeiss stemi 508; Oberkochen, Germany), according to the Blackman and Eastop [35] taxonomic key. During each sampling period, at least 5 adult specimens were collected from each area, apart from Agrokipio (due to the very low aphid population). These specimens were preserved in 98% ethanol at −80 °C for subsequent analysis.

### 2.2. Molecular Identification of Aphid Species

Five individual aphids collected from each different sampling area and period were used for the molecular analysis aiming at the species determination. Genomic DNA (gDNA) was extracted from single aphids using the DNeasy Blood and Tissue kit (Qiagen, Hilden, Germany) according to the manufacturer‘s protocol. Negative control (Nuclease-Free water; ThermoFisher/Invitrogen, CA, USA) was included in each DNA extraction series.

The mitochondrial Cytochrome Oxidase I (*COI*) gene was selected as the most suitable molecular marker for the discrimination of the aphid species by PCR, utilizing the universal primers LCO-1490 and HCO-2198 to amplify a 709 bp fragment [36]. A DNA template of 500 ng was employed, along with positive and negative controls (Nuclease-Free water; ThermoFisher/Invitrogen, CA, USA).

### 2.3. A Survey in CTV-Infected Fields, Virus Sources

In addition to the aphid species collected from the pilot citrus orchards, further surveys were conducted in fields where CTV has been continuously detected as part of a multiannual national project of CTV monitoring in citrus trees in Crete. These surveys were carried out during the autumn of 2021 and the spring and autumn of 2022 in the Vatolakkos area, where CTV has been established as an endemic form [27]. A total of 145 aphids were collected from 23 CTV-infected trees identified during the period from 2020 to 2022. *A. spiraecola* comprised the majority, with 137 individuals (93% of the total aphids), while *A. gossypii* and *T. aurantii* were each represented by an equal number of individuals (4 aphids each, 3% of the total) (Supplementary Table S1).

Plant material of young twigs with leaves collected from each quarter of two known labeled CTV-infected trees with either the European strain or the non-European stem-pitting strain, which was recently reported in Crete [27], served as positive controls in the molecular detection assays.

### 2.4. RNA Isolation from Plants and Aphids

A composite sample of leaves, petioles, and bark was collected from the four sub-samples from each quarter of the trees within the pilot orchards. Total RNAs were then isolated from 0.1 g of ground tissue using the TriZol method, as previously described [37]. The total RNAs were quantified using the Q5000 UV-Vis spectrophotometer (Quawell, Sunnyvale, CA, USA) and subsequently diluted to a concentration of 130 ng/μL for the molecular detection methods.

Two methods were evaluated for the isolation of total RNAs from the aphid species: the TriZol method and the Plant/Fungi Total RNA Purification kit (Norgen Biotek, Thorold, ON, Canada), both performed according to the manufacturer’s instructions. Initially, individual aphid adults or a pool of three adults, in cases where higher numbers were collected from a tree, were homogenized in a 1.5 mL tube using a pestle under liquid nitrogen. For the TriZol method, the following modifications were implemented: 500 μL TriZol buffer was used, the initial centrifugation step was omitted, 500 μL of ethanol was used in the wash step, and final resuspension in 15 μL of RNase-free water. Total RNAs were extracted only from adult alatae as there is no difference reported in acquisition ability from the apterous morphs [38].

### 2.5. CTV Detection in Aphids by RT-PCR or Real-Time PCR

Preliminary tests were performed to validate the RNA extraction procedure and assess the quality and integrity of the extracted RNA samples. For this purpose, a one-step RT-PCR was performed using 100 ng of total RNAs extracted from an individual or a pool of three adults, along with different primer pairs targeting three endogenous genes: LCO-1490 and HCO-2198 for the amplification of the *COI* gene [36], Mq-Fw and Mq-Rv for the Ribosomal 18S (*18S rRNA*) gene [39], and TMFw237 and TMRv461 for the Tropo-myosin (*TPM*) gene [39]. A common assay profile was used for the detection of all three genes. Briefly, the 25 µL RT-PCR reaction mix contained Green-Go Taq Flexi buffer (Promega, Madison, WI, USA), 1.5 mM MgCl_2_, 5 mM DTT, 0.25 mM dNTPs, 0.3 µM of each primer, 6 U RNase Inhibitor (NEB, Hitchin, UK), 1.25 U MML-V (Minotech, Crete, Greece), and 1.25 U Go-Taq polymerase (Promega, Madison, WI, USA), under the following cycling scheme: 50 °C for 60 min, 95 °C for 10 min, 40 cycles of 94 °C for 30 s, 60 °C for 30 s, 72 °C for 30 s, and a final step of 72 °C for 7 min; whereas for the *COI* gene, we had a modification with 5 cycles of 94 °C for 30 s, 51 °C for 30 s, 72 °C for 40 s, and 35 cycles of 94 °C for 30 s, 60 °C for 30 s, 72 °C for 40 s.

For the CTV detection in aphids and plants (served as positive controls), a conventional one-tube RT-PCR was performed using the CTV-1 and CTV-10 primers, which amplify the coat protein (*CP*) gene [40], as previously described [41]. From the aphids’ survey, 52 samples of a total of 127 randomly selected aphids were tested, representing all the aphid species identified during the three sampling periods and the four citrus hosts from the different pilot orchards (Supplementary Table S2). In addition, the 145 aphids collected from the CTV-infected fields were tested for the presence of the virus using the total RNAs extracted from individual alate adults only (Supplementary Table S1).

In cases where aphid samples were initially tested negative by one-tube RT-PCR, qPCR assays were employed as an additional diagnostic tool with enhanced sensitivity, ensuring that samples with low viral titers were not overlooked. The cDNAs were generated using 250 ng of total RNA from the same aphid samples and CTV-positive control plant samples (representing two different CTV genotypes), along with a negative control (healthy plant) and a non-template control (NTC) in a 10 μL reaction. A random hexamer primer and MML-V (Minotech, Crete, Greece) transcriptase were used according to the manufacturer’s instructions. For the qPCR, the protocol utilizing primers targeting a part of the *CP* gene was performed as described by Shegani et al. [42]. The reactions were carried out using the SYBR Green technology (KAPA Biosystems, Wilmington, MA, USA) according to the manufacturer’s instructions on a Real-Time Rotor-Gene 6000 system (Corbett/Qiagen, Hilden, Germany). The positive controls were also used in 2-, 5-, and 10-fold dilutions to determine the detection efficiency.

### 2.6. Sequence Analysis

The PCR products resulting from the *COI* gene amplification, used for aphid species discrimination, were purified with the NucleoFast 96 Vacuum Manifold Kit (Macherey-Nagel, Düren, Germany) according to the manufacturer’s instructions. Sequencing was performed in both directions using the same primers as those used for amplification (Macrogen, Amsterdam, The Netherlands). The obtained forward and reverse sequences of the amplified *COI* fragment were inspected and edited using the Geneious Prime 2023.0.1 bioinformatics software platform (https://www.geneious.com/ (accessed on 9 September 2024)). These sequences were then aligned against published sequences through the BLAST algorithm of the National Center for Biotechnology Information (NCBI, http://www.ncbi.nlm.nih.gov, accessed on 1 March 2024) for aphid species discrimination.

Two aphid isolates from *A. spiraecola* (isolate 20115) and *T. aurantii* (isolate 20781) were selected to confirm the viral origin of CTV. The RT-PCR amplicons raised from CTV detection in these aphids were purified using a column gel-extraction system (Macherey-Nagel, Düren, Germany) according to the manufacturer’s instructions. The purified DNA amplicons were Sanger sequenced in both orientations using the CTV-1 and CTV-10 primers (Macrogen, Amsterdam, The Netherlands). The nucleotide sequences were then compared with the available sequences in the GenBank database using the Blast-n software (version BLAST+ 2.15.0, accessed on 2 February 2024).

### 2.7. Wingbeat Analysis of Alate Adult Aphids

Optical wingbeat analysis methods typically rely on an emitter of infrared light that illuminates a volume of space (also called field of view—FOV) and a photodiode receiver that receives either variation of the cast shadow due to the presence of the flying insect or the backscattered light originating from the main body and wings of the flying insect [30]. Herein, an insect species identification based on their wingbeat was developed, which involves analyzing the unique light patterns created by the movement of an insect’s wings that modulate light. The optical means offer various advantages compared to acoustic monitoring using microphones. Small insects like aphids are hardly audible, and their recording would require specialized acoustic chambers, whereas optical wingbeat recorders are inherently immune to background audio interference.

**Figure 2:**
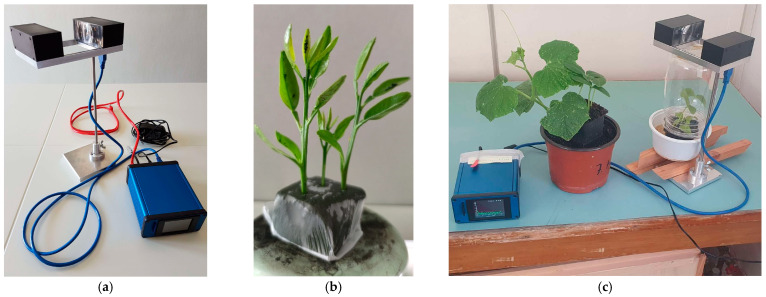
(**a**) An insects’ wingbeat recorder (the sensor on the left is the emitter–receiver pair while the blue box is the electronic board of the recorder; (**b**) Citrus stems infested by aphids; (**c**) A similar setup used for a preliminary study involving non-citrus plants and (alate) adult aphids.

In Figure 2a, we depict an experimental setup that we used to register wingbeat recordings of two common aphid species whose wingbeat characteristics are not sufficiently studied or are still pending in the literature. The device is based on a coupled emitter and receiver of infrared light that forms a ‘light blanket’. The aphids were manually collected and placed in a transparent jar containing a citrus plant, with an infrared light transmitter and receiver positioned outside the jar. The infrared light passed through the transparent container, capturing fluctuations caused by aphid takeoff. Since aphids are strongly attracted to yellow, yellow boards in various configurations were also utilized. As the aphids fly through the field of view, they modulate light with their wingbeat, which triggers the recorder to register the light fluctuations in the receiver (i.e., the casted shadow). The light variations are normalized and turned into an audible recording at an 8 kHz sampling rate that is subsequently stored in the SD card of the device for further processing. About 1000 (alate) adult aphids have been collected manually from the field using the setup in Figure 2c, in which we collected recordings from the species *A. spiraecola* and *A. gossypii* (Sternorrhyncha: *Aphididae*) feeding on tender citrus stems.

After identifying the most abundant aphid species and the predominant putative viruliferous CTV vector, we selected two species for wingbeat recording based on their importance and abundance. Manual identification and handling of insects are not scalable to a larger number of species and specimens, as this process requires collecting a vast number of manually identified cases that must pass through the emitter-receiver pair. Once the recordings are obtained, we apply state-of-the-art signal processing and machine learning algorithms that analyze the fundamental frequencies and harmonics of the wingbeat signals to automatically classify the two species. Machine learning techniques are trainable procedures where a suitable form of the recording is presented to the model along with its label (i.e., class attribution), and the error is propagated back to the system to adjust itself so that, in time, it makes smaller mistakes in its final classification. In the realm of deep learning architectures, as applied to audio, optoacoustic, and vibratory signals, the utilization of spectrograms and power spectral density (PSD) has emerged as the basic feature-extraction layer for various models.

Deep learning (DL) architectures have a modular layer composition where the layers close to the input learn to extract low-level features, and subsequent layers rely on the previous ones to progressively synthesize more complex representations that end in insect species labels.

Spectrograms are based on the short-time Fourier transform and provide a visual representation of the spectrum of frequencies of a signal as it varies with time, whereas the analysis of PSD reveals the distribution of power across different frequency components in a signal.

While the device plays a crucial role in our study, a detailed discussion of its electronic components and technical specifications falls outside the scope of this study, which focuses primarily on the biological and ecological aspects of the research. However, the engineering and technological underpinnings of the device can be found in the specialized literature cited in [30,33], as well as in a related application detailed in [34].

### 2.8. Statistical Analysis

Pairwise comparisons by Student’s *t*-test were used to compare the number of *A. spiraecola* compared to *A. gossypii* adults (alate and apterous forms) across citrus species, aiming to illustrate aphid composition in all pilot areas (Ayia, Vatolakkos, Apokoronas, Agrokipio). The effect of citrus and aphid species on the number of aphid adults collected in citrus stems was analyzed with two-way ANOVA for the same area and period separately. Similarly, the effect of citrus species and period on the number of nymphs collected in the same citrus stems was analyzed with two-way ANOVA (Ayia, Vatolakkos) and one-way ANOVA (Agrokipio, Apokoronas) for each period separately. The number of adult or nymph aphids was transformed to ln (x + 1) to stabilize variances. When significant, means were separated with the Tukey HSD test (α = 0.05). The analyses were carried out using the statistical package JMP Pro 13.2.1 [43].

## 3. Results

### 3.1. Aphids’ Composition and Population Distribution Among Citrus Species

Citrus shoots infested with aphids were collected from all pilot orchards to record the composition of aphid (alate and apterous forms of adult) species in the citrus-cultivated areas. Sampling of infested shoots among grapefruits, lemons, mandarins, and orange trees conducted in the Ayia area revealed two prevalent species out of five. *A. spiraecola* exhibited a mean percentage ranging from 44% (lemons in spring 2021) to 97% (mandarins in autumn 2021), making it one of the most frequently encountered aphid species. *A. gossypii* was also common, with a mean percentage ranging from 3% to 56%, although its abundance was not significantly higher than that of *A. spiraecola*. In addition to these two species, *T. aurantii, M. persicae* (Sulzer), and *A. craccivora* Koch were also detected, though in low numbers. In the Vatolakkos area, analysis of aphid data revealed that *A. spiraecola* was significantly more abundant than *A. gossypii*, with mean percentages ranging from 57% (grapefruit in autumn 2021) to 100% (orange trees in autumn 2022). An exception occurred in spring 2022 when *A. gossypii* accounted for 82% of the aphid population on infested grapefruit stems. In the Agrokipio orange orchard, a noticeable but low aphid population was recorded compared to lemon and mandarin orchards in the same area, in which only a limited number of tender shoots were observed and subsequently infested by aphids. Similarly, in the Apokoronas (Stylos) area, low aphid populations were collected across the tested periods, possibly due to reduced cultural practices. Specifically, in the Apokoronas (Stylos) area, *A. spiraecola* ranged from 76 to 98%, while in Agrokipio, from 29% (mandarins in spring 2021) to 99% (oranges in spring 2022) across all tested periods, confirming its status as one of the most frequent aphid species in these areas (Supplementary Table S3).

**Figure 3:**
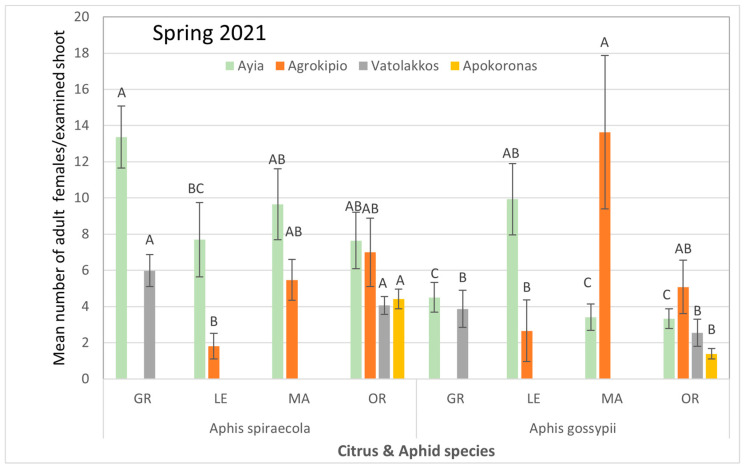
Mean number ± SE of adult (alate and apterous) aphid population per citrus and aphid species for each selected region during spring of 2021. Bars of the same color (interaction of aphid and citrus species of the same region and season) followed by different letters are significantly different at *p* < 0.05. GR: grapefruit, LE: lemon, MA: mandarin, OR: orange.

Throughout all tested areas and sampling periods, it was primarily the aphid species that influenced aphid colonization on citrus species rather than the citrus species themselves (Supplementary Table S4). *A. gossypii* typically appeared early in the summer but was subsequently significantly reduced with the appearance of *A. spiraecola*. Moreover, in the Ayia area, analyses of infested shoots in spring 2021 revealed a significantly higher average count of *A. spiraecola* in grapefruits compared to populations of *A. gossypii* collected from all citrus species. Conversely, in Agrokipio, *A. gossypii* exhibited significantly higher populations on mandarins compared to *A. spiraecola* counted on infested lemon trees, while aphid species populations on other citrus species showed no significant differences among them. In Vatolakkos, *A. spiraecola* preferred tender stems of oranges over those of other citrus species. In Apokoronas, where orange trees are the primary citrus crop, the average population of *A. spiraecola* was significantly higher than that of *A. gossypii* (Figure 3).

**Figure 4:**
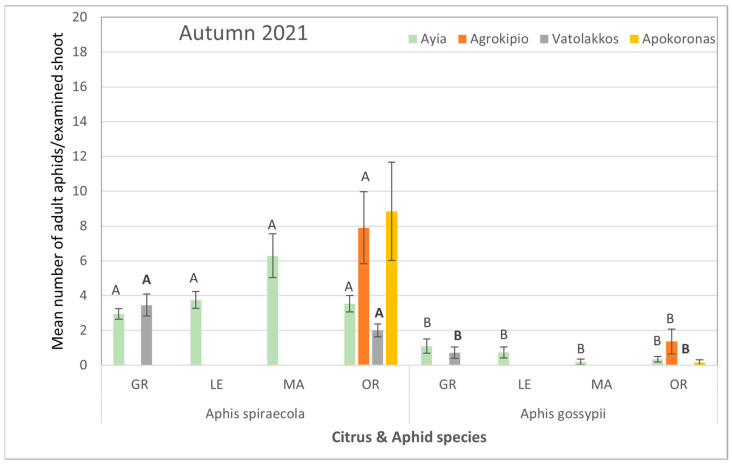
Mean number ± SE of adult aphid (alate and apterous) population per citrus and aphid species for each selected region during autumn of 2021. Bars of the same color (interaction of aphid and citrus species of the same region and season) followed by different letters are significantly different at *p* < 0.05. GR: grapefruit, LE: lemon, MA: mandarin, OR: orange.

During the autumn of the same year, although the aphid colonization of citrus plants was reduced, the aphid species themselves rather than the citrus host plant had a significant impact on population levels. Specifically, the average counts of *A. spiraecola* collected from all tested areas were significantly higher than those of *A. gossypii* (Supplementary Table S4, Figure 4).

**Figure 5:**
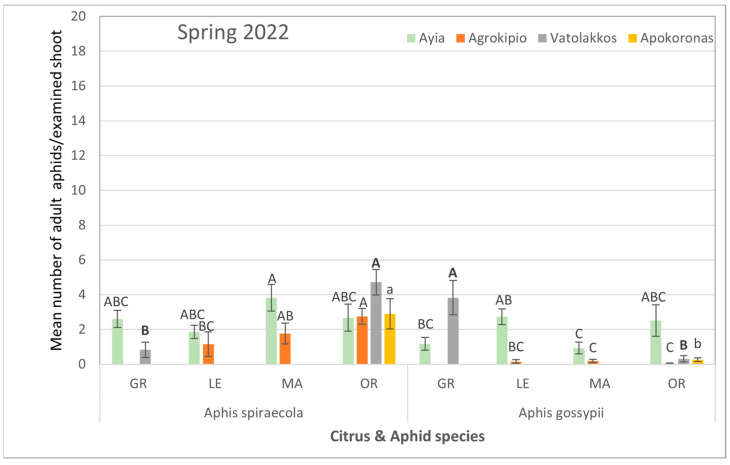
Mean number ± SE of adult aphid (alate and apterous) population per citrus and aphid species for each selected region during spring of 2022. Bars of the same color (interaction of aphid and citrus species of the same region and season) followed by different letters are significantly different at *p* < 0.05. GR: grapefruit, LE: lemon, MA: mandarin, OR: orange.

Similarly, spring samplings in 2022 indicated a dominance of *A. spiraecola* in mandarin trees of Ayia and in orange trees of Agrokipio, Vatolakkos, and Apokoronas over *A. gossypii* colonization among citrus species (Supplementary Table S4, Figure 5).

**Figure 6:**
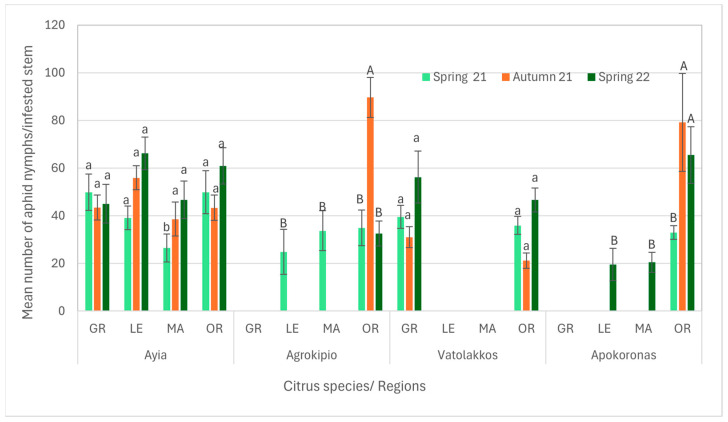
Mean number ± SE of aphid nymphs’ population per citrus species and season of the same region. Bars of each citrus species and season for the same region followed by different small or capital letters are significantly different at *p* < 0.05. GR: grapefruit, LE: lemon, MA: mandarin, OR: orange.

Regarding aphid nymph populations across all infested citrus stems and periods, the Ayia area showed that the nymph population was influenced by citrus species, period as well as their interaction. Notably, mandarin trees exhibited a significantly lower nymph population during spring 2021 compared to other citrus species. Conversely, in the Vatolakkos area, nymph populations were higher on grapefruit and orange stems recorded during spring 2022 compared to other citrus stems across all periods. In the Agrokipio area, nymph populations on orange stems during autumn 2021 were significantly higher than in other cases. Similarly, in the Apokoronas area, nymphs’ colonization was significantly higher on orange stems during both autumn 2021 and spring 2022 (Supplementary Table S5, Figure 6).

### 3.2. Molecular Identification of Aphid Species

All aphid individuals morphologically identified as *A. spiraecola* or *A. gossypii* were also confirmed through PCR amplification of the *COI* gene and sequencing of the resultant amplicon. Sequence comparison with homologous sequences available in the NCBI database resulted in 100% similarity to either *A. gossypii* or *A. spiraecola* across all dates and sampling areas. Two representative sequences have been submitted to the NCBI database under the accession numbers PQ305555 and PQ305556 for *A. gossypii* and *A. spiraecola*, respectively.

### 3.3. Evaluation of the Total RNA Isolation from Aphids

Both the TriZol method and the commercial kit yielded total RNA extracts from aphids with comparatively high quality (A260/280 values were ±0.2) and satisfactory concentrations (from 70 to 320 ng/μL). Amplification of *COI* and *18S rRNA* genes was successful by both methods, confirming the integrity of the recovered total RNAs. Consequently, the TriZol method was used for subsequent analyses. The commercial kit was employed restricted only in cases where RNA recovery was problematic with the TriZol method. It is worth noting that the amplification of the *TPM* gene was inconsistent, potentially due to mismatches between the primer sequences and the aphid *TPM* gene sequence, as the primers were designed based on leafhopper species.

### 3.4. Identification of Aphids as Putative Vectors of CTV in This Study

None of the 52 aphid samples (composed of 127 individuals) collected from the pilot citrus orchards, nor the citrus trees themselves, tested positive for CTV. To exclude the possibility of RNA degradation inhibiting an accurate detection, an RT-PCR assay using primers for the *18S rRNA* gene was performed on 20 random RNA samples out of the 52. Successful amplification was achieved in all cases.

The analysis of aphids collected from the CTV-infected fields initially revealed the presence of RT-PCR-positive aphids for CTV in 20 out of the 23 infected trees; these are hereafter referred to as putative viruliferous aphids. The CTV was detected in 95 aphids by conventional RT-PCR. In the case of three trees (20134, 20111, 21820) from which no CTV-positive aphids were detected, a conventional RT-PCR was performed on plant tissue (leaves from which the aphids were collected). These results confirmed the presence of CTV in the plant tissue, excluding the possibility of collecting by default tissue from a CTV-free part of the tree, as CTV is known to have an uneven distribution within infected trees.

The analysis of 35 random samples from aphids and plants collected from the pilot orchards by qPCR confirmed them as negative. Fifty aphid samples from the CTV-infected fields that were not found positive for CTV by conventional RT-PCR (Supplementary Table S1) were also tested by qPCR. These results revealed two additional putative viruliferous aphids from the trees 20134 and 21820.

In summary, no aphids collected from the pilot citrus orchards tested positive for CTV. However, from the CTV-infected fields, 97 out of 145 aphids from 22 out of 23 trees were identified as putative viruliferous aphids. The predominant putative viruliferous aphid species was *A. spiraecola*, accounting for 93.81% (91 out of 97 putative viruliferous aphids), followed by *A. gossypii* at 4.12% (4 out of 97 putative viruliferous aphids) and *T. aurantii* at 2.06% (2 out of 97 putative viruliferous aphids) (Supplementary Figure S1).

### 3.5. CTV Sequences from Putative Viruliferous Aphids

The sequence analysis confirmed the virus origin of CTV from both aphid species, each 672 nt in length. These CTV sequences from aphids also represent the first such sequences available from these vectors in Greece. The two CTV sequences from aphids were identical and shared 99% of identity with isolate KF908013 from Crete (Greece) and 99.9% identity with isolate OP820240 from China, both of which belong to the RB genotype. The sequences used in this study were deposited in GenBank under accession numbers PQ824256 and PQ824257.

### 3.6. Wingbeat Analysis of Alate Adult Aphids

**Figure 7:**
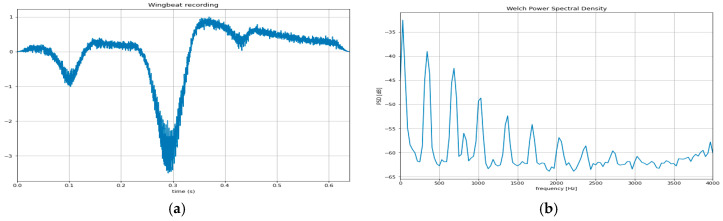
(**a**) A typical wingbeat recording of an adult alate aphid in flight; (**b**) the power spectral density of the signal reveals its frequency content. In which frequencies the peaks lie and their corresponding amplitude constitutes a unique ‘recipe,’ also referred to as ‘spectral signature’, which is unique for each species.

About 1000 recordings of alate adult aphids have been collected manually in the setting of Figure 7b. The species *A. spiraecola* and *A. gossypii* feeding on tender citrus stems were studied. A random sample of 70% of the recordings is kept as a standard of training, and the remaining 30% is the test set. Note that one individual is responsible for each recording; therefore, there is less than 1% of recordings originating from the same individual (Table 2).

The distribution of energy over frequencies is derived from all recordings (Figure 7b). Several learning algorithms based on Convolutional Neural Networks, such as DenseNet121, InceptionV3, MobileNet, and NASNetMobile (Table 2), are trained in the training set and evaluated on the test set.

These architectures showcase an impressive range of accuracies ranging from 95.2% to 95.6%. On the other hand, the analysis of PSD, which reveals the distribution of power across different frequency components in a signal, has found prominence in architectures like 5-layer CNN, attaining an accuracy of 93.3%. These models underscore the importance of leveraging different types of signal representations for optimal performance in various tasks. Additionally, in the domain of shallow learners, models like XGBoost and LightGBM have demonstrated respectable accuracies of 87.1% and 86.4%, respectively, by employing PSD analysis. This amalgamation of advanced deep learning architectures alongside traditional shallow learners signifies the diverse approaches taken in machine learning to address complex problems efficiently.

## 4. Discussion

*A. spiraecola* was the most abundant aphid species identified in citrus orchards of Crete, followed by *A. gossypii* as confirmed through both morphological and molecular methods. Additionally, *Τ. aurantii, M. persicae,* and *A. craccivora* were also recorded, though in very low numbers. These findings remained consistent until the end of the sampling period in spring 2022. Similarly, other studies have reported the *A. spiraecola* and *A. gossypii* as the most abundant species (45% and 40%, respectively) in addition to the identification of *T. aurantii* and *M. persicae* in lower populations in citrus groves in Italy (Apulia) [44]. In Spain, *A. gossypii* was found in 53% and *A. spiraecola* in 32% of the samples, with *T. aurantii* (11%), *M. persicae* (1%), and *A. craccivora* (1%) also present [10]. In contrast, during a 3-year survey in Portugal, it was noticed that *A. spiraecola* was the most predominant species (79–95%), while *A. gossypii* was found in much lower percentages (4–20%), along with *A. aurantii* and *Macrosiphum euphorbiae* [45]. However, in India, five aphid species were identified in citrus groves, with *A. citricidus* being the most abundant and responsible for the maximum spread of CTV [46].

A key finding was that citrus species did not significantly influence aphid colonization in most of the sampled areas. It was obvious that aphid colonization was evidently influenced by the vegetative growth and vigor of citrus trees, which are also affected by cultural practices such as pruning, fertilization, and irrigation. Vegetative growth was generally limited across all examined citrus orchards, notably impacting aphid aggregations. On these tender shoots, *A. gossypii* typically appeared early in the summer, followed by a significant reduction in its population with the subsequent emergence of *A. spiraecola*, which eventually became the dominant aphid species, as similarly reported by Kalaitzaki et al. [17] from previous surveys during 2016 and 2017. As *A. spiraecola* becomes more abundant, it outcompetes *A. gossypii* for resources such as food, mating partners, and suitable habitats. This competition leads to a decline in the population of *A. gossypii*, as it is less able to establish and maintain its numbers in the presence of *A. spiraecola*. The subsequent population decline due to the appearance of *A. spiraecola* is a direct result of this interspecific competition. The dynamics of aphids in citrus orchards can also be influenced by responses of citrus trees from nitrogen fertilization, with *A. spiraecola* being the most abundant among species, comprising 84–94.55% of the aphid population [47].

It is noteworthy that *A. citricidus*, globally referred to as the most efficient CTV vector, has not been detected in citrus orchards of Chania. This trend remained consistent throughout multiple years of observations. The absence of *A. citricidus* in Greece may be attributed to several factors, potentially including its non-native status in the Mediterranean region [48]. Although *A. citricidus* was discovered in Portugal and parts of northern Spain in the early 2000s, there is currently no evidence of its spread to other regions and countries within the Mediterranean basin.

Studies of CTV in Greece have been primarily focused on the detection and characterization of three genotypes [27,28,29] during a multiannual national monitoring program aimed at identifying new infection loci. Field reports on aphid species potentially serving as efficient CTV vectors in Greece or other countries within local micro-enviroments are limited, except for in vitro transmission tests [14]. The use of the Trizol method for RNA extraction from aphids was effective in retrieving good quality, maximizing the accuracy of CTV diagnosis in aphids, in accordance with previous reports [49]. Our results indicate the existence of three aphid putative viruliferous species, with *A. spiraecola* being the most dominant (93.8%) carrying the CTV in the total number of aphid putative viruliferous adults, followed by *A. gossypii* and *T. aurantii*. Although the percentages of CTV-infected aphids were higher in *A. gossypii* (100%; four out of four) compared to *A. spiraecola* (66.4%, 91 out of 137) relative to their respective population, the overall population of *A. spiraecola* found in CTV-infected trees was higher, in accordance with the above data of its population dynamics observed in the pilot orchards. Furthermore, putative viruliferous *A. gossypii* and *T. aurantii* species were observed in the same trees where CTV-infected *A. spiraecola* adults were also detected. On the contrary, the ratio of viruliferous *A. gossypii* and *A. spiraecola* in Spain were similar (27% and 23%, respectively), which was also in accordance with their population dynamics [10], whereas in Portugal, although these two aphid species exhibited significant differences in their population dynamics, they were found to carry CTV at similar percentages [44].

*A. gossypii* has been reported as the second most efficient vector during transmission experiments [14], and in this study, we observed that its adaptation in Crete appears to be less abundant compared to the dominant *A. spiraecola* potentially due to environmental factors or insecticide resistance/sensitivity. Overall, these data make us strongly hypothesize that *A. spiraecola* may be the main important vector of CTV in Crete due to its abundance over *A. gossypii* and in the absence of *A. citricidus*. Similarly, although the proportion of detected viruliferous *A. spiraecola* adults was lower than that of *A. gossypii* (35% vs. 29%), their population dynamics were vice versa, leading to *A. spiraecola* being considered as the main vector for the CTV spread in Morocco [50]. It has been reported that the CTV genotype populations can affect the virus transmission by aphids [51], and in Crete, the T30 genotype has been overtaken by the RB genotype, which could also explain the different transmission between species. However, further studies are needed to confirm this hypothesis.

The efficient control of CTV relies on the use of certified propagating material in addition to CTV-resistance rootstocks and the management of aphid population outbreaks in CTV-infected areas to prevent its wider spread [14]. Recently, novel technologies using remote sensing methods have been developed for accurate and early detection of CTV as a control strategy [41,52]; however, to our knowledge, no related studies have been conducted to monitor aphids in relation to their role as CTV vectors. During the last decade, numerous reports have reported the development of electronic traps [31,53,54,55] and various sensors, such as Lidars [56], for insect classification and monitoring. In the case of aphids, research on flying patterns remains limited, likely due to their small size and distinctive flying habits [57]. Regarding operation in the field where sensors operate in unconstrained conditions, wind can influence the harmonic coefficients of an insect’s wingbeat but not its fundamental frequency. In this work, we describe the development and implementation of an electronic sensor to assess the discrimination of the two main aphid species identified in Crete, which were also found as putative vectors of CTV, based on the laboratory wingbeat analysis. Our results report their successful classification and are in accordance with the findings in [34], where optical wingbeat recordings were used for the discrimination of different aphid species. Nevertheless, in our study, the classification accuracy reached ~95%, significantly higher than the study of *A. fabae* and three other aphids [34] and similar to the one reported for the *A. gossypii* identification (94%) using an artificial neural network [58].

Wingbeat analysis relies on factors such as variations in wing morphology and wingbeat behavior that are being picked up by data-driven machine learning techniques and affect the accuracy of identification. Overall, while insect species recognition based on wingbeat patterns has its advantages and can be accurate in certain contexts, it is not as precise as molecular differentiation based on *COI* gene analysis by PCR or taxonomic identification by experts using keys, particularly in situations requiring high specificity or when dealing with closely related species. However, it remains a low-cost, reliable approach capable of delivering real-time data with sufficient accuracy for certain applications, such as tracking pest dynamics in the field. Enlarged versions of arrays of low-cost sensors are also suitable for integration into moving platforms such as tractors and drones to support situation awareness in the field regarding a targeted or limited number of flying aphids counting in real-time, which is the end goal of future research.

## 5. Conclusions

This study provides comprehensive insights into the aphid species composition and their role as potential vectors of CTV in citrus orchards of Crete. The most abundant aphid species identified were *A. spiraecola* and *A. gossypii*, with the former emerging as the dominant species over time, reaching up to 100% in a few cases regardless of the citrus species.

Our findings also highlight the primary putative vector for CTV in Crete, particularly in the absence of the highly efficient *A. citricidus*. The use of molecular methods, including RNA extraction via the Trizol method, proved effective for the accurate detection of CTV in aphid species. While *A. gossypii* exhibited higher percentages of putative viruliferous individuals in relation to its population, the dominance of *A. spiraecola* in infected trees reinforces its role as the main important CTV vector in the region.

The development and implementation of novel technologies, such as electronic traps utilizing wingbeat analysis and machine learning, offer promising avenues for improving aphid monitoring, as demonstrated herein. Future research should focus on enhancing these technologies (e.g., e-traps adapted to drones scanning a citrus orchard) and exploring the potential effects of CTV genotype variability on virus transmission dynamics to strengthen citrus orchard management practices.

## Figures and Tables

**Table 1:** Dates of sampling of infested shoots with aphids, citrus species, and locations of each orchard.

Area	Co-Ordinates	No of Orchards	Citrus Species	Date of Sampling During Autumn 2020, Spring and Autumn 2021, Spring 2022
Ayia	35.4678223, 23.9202723	4	Grapefruit, Lemon, Mandarin, Orange	2020: 5.11
	35.4763655, 23.9419056 35.4715441, 23.9257644			2021: 7.4, 19.4, 4.5, 14.5, 27.5, 7.6, 22.6, 10.9, 22.9, 4.10, 15.10, 27.10, 8.11
	35.4680203, 23.9196292			2022: 19.4, 3.5, 16.5, 26.5, 7.6
Vatolakkos	35.4564375, 23.8971727 35.4519540, 23.8864000	2	Grapefruit, Orange	2020: 5.11 2021: 14.5, 27.5, 7.6, 22.6, 10.9, 22.9, 4.10, 15.10, 27.10, 8.11 2022: 19.4, 3.5, 16.5, 26.5, 7.6
Apokoronas (Stylos)	35.4479499, 24.1353730	3	Orange	2021: 7.4, 9.4, 4.5, 14.5, 27.5, 7.6, 22.6, 10.9, 22.9, 4.10, 15.10, 27.10, 8.11
	35.4464094, 24.1386021			
	35.4383835, 24.1322942			2022: 19.4, 3.5, 16.5, 26.5, 7.6
Agrokipio	35.4863107, 24.0485113	1	Lemon, Mandarin, Orange	2021: 14.5, 27.5, 7.6, 22.6 10.9, 22.9, 4.10, 15.10, 27.10, 8.11 2022: 19.4, 3.5, 16.5, 26.5

**Table 2:** Deep learning approaches for classifying the wingbeat of aphids.

Model	Features	(%) Accuracy
DenseNet121	Spectrogram	95.6
5-layer CNN	PSD	93.3
InceptionV3	Spectrogram	95.3
MobileNet	Spectrogram	95.2
Xception	Spectrogram	93.9
NASNetMobile	Spectrogram	94.1
**Shallow Learners**		
XGBoost	PSD	87.1
LightGBM	PSD	86.4

## Data Availability

The data presented in this study are available upon request from the corresponding author.

## Supplementary Materials

The following supporting information can be downloaded at https://www.mdpi.com/article/10.3390/v17030395/s1. Figure S1: Bar graph showing the number of CTV-infected trees used in this study and the number of infected trees in which CTV-viruliferous aphids were detected. The last bars show the total number of the aphids tested and the number of CTV-viruliferous aphids per species; Table S1: Aphid sampling from CTV-infected trees and detection of CTV in aphids by conventional RT-PCR and qPCR; Table S2: Selected aphid species, collected from the four pilot citrus orchards during the study of their population variation in different hosts, for molecular detection of CTV. Table S3: Percentage of aphid species (*Aphis spiraecola* vs. *Aphis gossypii*) distribution in orchards of Chania across seasons 2021 and 2022 (paired *t*-test). Table S4: The effect of citrus and aphid species on aphid adult populations collected from citrus stems of four regions in Crete across seasons 2021 and 2022 (2-way ANOVA). Table S5: The effect of citrus species and season on aphid nymph populations collected from citrus stems of four regions in Crete across seasons 2021 and 2022.
