# Supplementary material for: Aphid Species in Citrus Orchards in Crete: Key Vectors of Citrus Tristeza Virus and Automated Monitoring Innovations for Alate Aphids
Source: Viruses. 2025 Mar 11;17(3):395. doi: 10.3390/v17030395 (PMC11946668; doi:10.3390/v17030395)
Supplement: Supplementary file 1 [file viruses-17-00395-s001.zip › Supplementary Tables S1-S5.pdf]

Supplementary Table S1. Aphid sampling from CTV-infected trees and detection of CTV in aphids by conventional RT-PCR and qPCR.

Sampling period	Tree Code	Aphid species	Number of collected adult aphids	Number of CTV positive samples by RT-PCR	Number of samples repeated by qPCR / number of CTV positive samples
**Autumn 2021**	20140	*A. spiraecola*	7	4	3 / 0
	20110	*A. spiraecola*	2	1	1 / 0
	20143	*A. spiraecola*	2	2	-
	20134	*A. spiraecola*	1	0	1 / 1
	20115	*A. spiraecola*	2	1	1 / 0
	20108	*A. spiraecola*	11	5	6 / 0
	20137	*A. spiraecola*	1	1	-
	20111	*A. spiraecola*	3	0	3 / 0
	20609	*A. spiraecola*	4	3	1 / 0
	20560	*A. spiraecola*	6	4	2 / 0
		A. gossypii	3	3	-
**Spring 2022**	20609	*A. spiraecola*	4	2	2 / 0
	20781	*A. spiraecola*	8	3	5 / 0
		T. auranti	2	2	-
	20765	*A. spiraecola*	6	5	1 / 0
**Autumn 2022**	20781	*A. spiraecola*	7	1	6 / 0
		T. auranti	2	0	2 / 0
	20765	*A. spiraecola*	3	3	-
	22540	*A. spiraecola*	7	3	4 / 0
	21820	*A. spiraecola*	4	0	4 / 1
	21828	*A. spiraecola*	8	8	-
	21844	*A. spiraecola*	7	7	-
	21842	*A. spiraecola*	1	1	-
	21838	*A. spiraecola*	7	5	2 / 0
	22686	*A. spiraecola*	9	9	-
	21845	*A. spiraecola*	9	9	-
	22713	*A. spiraecola*	10	10	-
		A. gossypii	1	1	-
	21837	*A. spiraecola*	4	1	3 / 0
	22539	*A. spiraecola*	4	1	3 / 0
**Total**		*A. spiraecola*	137	89	2
		A. gossypii	4	4	0
		T. auranti	4	2	0

Supplementary S2. Selected aphid species, collected from the four pilot citrus orchards during the study of their population variation in different hosts, for molecular detection of CTV.

Host	Area	Tree Code	Aphid species	Number of adults per sample
**Grapefruit**	Ayia	87	*T. auranti*	3
	Ayia	181	*A. spiraecola*	3
	Ayia	247	*A. gossypii*	2
	Vatolakkos	110	*A. spiraecola*	3
	Vatolakkos	120	*A. gossypii*	3
	Ayia	293	*A. spiraecola*	3
	Ayia	310	*A. gossypii*	2
	Ayia	145	*A. gossypii*	1
	Ayia	164	*A. gossypii*	1
**Lemon**	Ayia	185	*A. spiraecola*	3
	Ayia	299	*A. gossypii*	3
	Ayia	300	*T. auranti*	2
	Agrokipio	276	*A. gossypii*	2
	Agrokipio	277	*T. auranti*	3
	Agrokipio	305	*A. spiraecola*	2
**Mandarin**	Agrokipio	249	*A. gossypii*	1
	Agrokipio	251	*T. auranti*	1
	Agrokipio	278	*A. spiraecola*	3
	Ayia	219	*A. craccivora*	3
	Ayia	266	*A. spiraecola*	3
	Ayia	267	*A. gossypii*	3
	Ayia	268	*T. auranti*	2
**Orange**	Ayia	116	*A. gossypii*	3
	Ayia	155	*A. spiraecola*	3
	Ayia	156	*A. gossypii*	3
	Ayia	160	*A. spiraecola*	3
	Ayia	239	*T. auranti*	2
	Ayia	240	*M. persicae*	1
	Agrokipio	146	*A. spiraecola*	3
	Agrokipio	172	*A. gossypii*	3
	Agrokipio	258	*M. persicae*	3
	Agrokipio	289	*T. auranti*	3
	Agrokipio	318	*A. spiraecola*	1
	Vatolakkos	135	*A. spiraecola*	3
	Vatolakkos	187	*A. spiraecola*	3
	Vatolakkos	209	*M. persicae*	1
	Vatolakkos	265	*T. auranti*	1
	Vatolakkos	308	*A. gossypii*	3
	Ayia	215	*A. spiraecola*	3
	Ayia	184	*A. gossypii*	2
	Ayia	195	*A. spiraecola*	3
	Ayia	271	*T. auranti*	1

Ayia	295	*A. gossypii*	3
Stylos	111	*A. spiraecola*	3
Stylos	122	*A. spiraecola*	3
Stylos	241	*A. gossypii*	3
Stylos	242	*A. spiraecola*	3
Stylos	243	*T. auranti*	3
Stylos	253	*T. auranti*	2
Stylos	254	*A. craccivora*	1
Stylos	302	*A. gossypii*	3
Stylos	303	*T. auranti*	3

Supplementary Table S3. Percentage of aphid species (*Aphis spiraecola* vs *Aphis gossypii)* distribution in orchards of Chania across seasons 2021 and 2022 (paired t-test).

Area	Citrus species	Spring 2021				Autumn 2021				Spring 2021			
		% *A. spiraecola*	df	t	p	% *A. spiraecola*	df	t	p	% *A. spiraecola*	df	t	p
**Ayia**	Oranges	68	112	2.26	**0.025**	91	100	6.128	**0.000**	81	198	3.343	**0.001**
	Grapefruits	78	111	4.423	**0.000**	73	103	3.233	**0.001**	71	103	1.811	0.073
	Mandarins	68	53	2.480	**0.016**	97	54	4.773	**0.000**	95	110	6.095	**0.000**
	Lemons	44	113	0.740	0.458	84	109	4.126	**0.000**	56	132	1.173	0.242
**Vatolakkos**	Oranges	62	92	1.701	0.091	100	39	5.325	**0.000**	94	49	5.406	**0.000**
	Grapefruits	64	63	2.302	**0.024**	57	47	1.100	0.276	18	17	2.395	**0.028**
**Agrokipio**	Oranges	53	23	0.357	0.723	85	18	3.717	**0.001**	99	47	6.147	**0.000**
	Mandarins	29	18	2.018	0.058	-	-	-	-	90	20	2.637	**0.015**
	Lemons	42	15	0.408	0.689	-	-	-	-	88	12	1.362	0.198
**Apokoronas**	Oranges	76	139	4.694	**0.000**	98	12	3.057	**0.009**	92	51	2.945	**0.004**

* The absence of values within table indicates a limited numbers of aphid individuals, rendering statistical analysis unfeasible.

Supplementary Table S4. The effect of citrus and aphid species on aphid adult populations collected from citrus stems of four regions in Crete across seasons 2021 and 2022 (2-way ANOVA).

**Area**	**Factor**	**Spring 2021**			**Autumn 2021**			**Spring 2022**		
		**df**	**F**	**p**	**df**	**F**	**p**	**df**	**F**	**p**
**Ayia**	Aphid species	1,946	31.816	**<0.0001**	1,723	340.79	**<0.0001**	1,380	8.476	**<0.0038**
	Citrus species	3,926	1.357	<0.254	3,715	0.276	<0.842	3,287	0.391	<0.7596
	Interaction	3,946	17.468	**<0.0001**	3,723	3.774	**<0.010**	3,380	6.421	**<0.0003**
**Vatolakkos**	Aphid species	1,305	47.075	**<0.0001**	1,167	79.411	**<0.0001**	1,126	1.773	<0.1854
	Citrus species	1,307	6.821	**<0.0094**	1,28	3.672	<0.065	1,100	0.043	<0.8354
	Interaction	1,305	0.370	<0.543	1,167	0.007	<0.931	1,100	52.233	**<0.0001**
**Agrokipio**	Aphid species	1,109	0.096	<0.757	-	-	-	1,11	34.87	**<0.0001**
	Citrus species	2,60	9.481	**<0.0003**	-	-	-	2,1	2.013	<0.137
	Interaction	2,109	1.101	**<0.336**	-	-	-	2,2	4.124	**<0.0179**
**Apokoronas**	Aphid species	1,272	46.852	**<0.0001**	1,19	28.523	**<0.0001**	1,97	21.456	**<0.0010**
	Citrus species	0	0	-	0	0	-	0	0	-
	Interaction	0	0	-	0	0	-	0	0	-

* The absence of values within table indicates a limited numbers of aphid individuals, rendering statistical analysis unfeasible.

** Ayia orchards: Grapefruit, Lemon, Mandarin, Orange, Vatolakkos orchards: Grapefruit, Orange, Apokoronas orchards: Orange, Agrokipio orchards: Lemons, Mandarins, Orange.

Supplementary Table S5. The effect of citrus species and season on aphid nymph populations collected from citrus stems of four regions in Crete across seasons 2021 and 2022.

	**Ayia** *(2-way ANOVA)*			**Vatolakkos** *(2-way ANOVA)*			**Agrokipio** *(1-way ANOVA)*		
**Factor**	**df**	**F**	**p**	**df**	**F**	**p**	**df**	**F**	**p**
Season	2,15	3.399	**<0.05**	2,6	4.853	<0.055	4,120	10.117	**<0.0001**
Citrus species	3,904	7.525	**<0.0001**	1,77	1.440	<0.233	**Apokoronas** (*1-way ANOVA)*		
Interaction	6,904	5.301	**<0.0001**	2,85	0.268	<0.765	4,234	6.312	**<0.0001**

* Ayia orchards: Grapefruit, Lemon, Mandarin, Orange, Vatolakkos orchards: Grapefruit, Orange, Apokoronas orchards: Orange, Agrokipio orchards: Lemons, Mandarins, Orange.
