# Supplementary figures and images for: Aphid Species in Citrus Orchards in Crete: Key Vectors of Citrus Tristeza Virus and Automated Monitoring Innovations for Alate Aphids
Source: Viruses. 2025 Mar 11;17(3):395. doi: 10.3390/v17030395 (PMC11946668; doi:10.3390/v17030395)

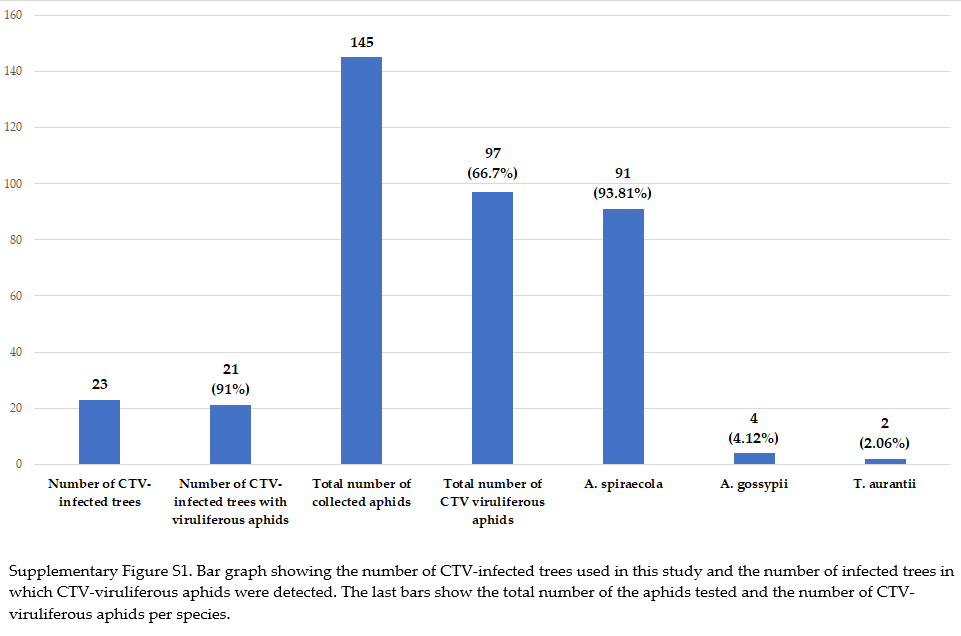

Supplement: Supplementary file 1 [file viruses-17-00395-s001.zip › Supplementary Figure S1.png]
